# Novel neuroblastoma amplified sequence (*NBAS*) mutations in a Japanese boy with fever-triggered recurrent acute liver failure

**DOI:** 10.1038/s41439-018-0035-5

**Published:** 2019-01-07

**Authors:** Sahoko Ono, Junko Matsuda, Etsuko Watanabe, Hiroto Akaike, Hideto Teranishi, Ippei Miyata, Takanobu Otomo, Yoshito Sadahira, Tatsuki Mizuochi, Hironori Kusano, Masayoshi Kage, Hiroo Ueno, Kenichi Yoshida, Yuichi Shiraishi, Kenichi Chiba, Hiroko Tanaka, Satoru Miyano, Seishi Ogawa, Yasuhide Hayashi, Hirokazu Kanegane, Kazunobu Ouchi

**Affiliations:** 1 Department of Pediatrics, Kawasaki Medical School, Okayama, 701-0192 Japan; 2 Department of Pathophysiology and Metabolism, Kawasaki Medical School, Okayama, 701-0192 Japan; 3 Department of Pathology, Kawasaki Medical School, Okayama, 701-0192 Japan; 4 Department of Pediatrics and Child Health, Kurume University School of Medicine, Kurume, 830-0011 Japan; 5 Department of Pathology, Kurume University School of Medicine, Kurume, 830-0011 Japan; 6 Research Center for Innovative Cancer Therapy, Kurume University School of Medicine, Kurume, 830-0011 Japan; 7 Department of Pathology and Tumor Biology, Graduate School of Medicine, Kyoto University, Kyoto, 606-8501 Japan; 8 Laboratory of DNA Information Analysis, Human Genome Center, Institute of Medical Science, The University of Tokyo, Tokyo, 108-8639 Japan; 9 Laboratory of Sequence Analysis, Human Genome Center, Institute of Medical Science, The University of Tokyo, Tokyo, 108-8639 Japan; 10 Institute of Physiology and Medicine, Jobu University, Gunma, 370-1393 Japan; 11 Department of Child Health and Development, Graduate School of Medical and Dental Sciences, Tokyo Medical and Dental University (TMDU), Bunkyo-ku, Tokyo 113-8519 Japan

**Keywords:** Medical genetics, Liver diseases

## Abstract

Biallelic mutations in the neuroblastoma amplified sequence (*NBAS*) gene have been reported to cause two different clinical spectra: short stature with optic nerve atrophy and Pelger-Huët anomaly (SOPH) syndrome and infantile liver failure syndrome 2 (ILFS2). Here, we describe a case of a 3-year-old Japanese boy who presented with fever-triggered recurrent acute liver failure (ALF). The clinical characteristics were considerable elevation of liver enzymes, severe coagulopathy, and acute renal failure. In addition to the liver phenotype, he had short stature and Pelger-Huët anomaly in the peripheral granulocytes. Whole-exome and Sanger sequencing of the patient and his parents revealed that he carried novel compound heterozygous missense mutations in *NBAS*, c.1018G>C (p.Gly340Arg) and c.2674 G>T (p.Val892Phe). Both mutations affect evolutionarily conserved amino acid residues and are predicted to be highly damaging. Immunoblot analysis of the patient’s skin fibroblasts showed a normal NBAS protein level but a reduced protein level of its interaction partner, p31, involved in Golgi-to-endoplasmic reticulum retrograde vesicular trafficking. We recommend *NBAS* gene analysis in children with unexplained fever-triggered recurrent ALF or liver dysfunction. Early antipyretic therapy may prevent further episodes of ALF.

## Liver disease: Mutations associated with recurrent liver failure

Novel mutations in a gene called *NBAS* have been identified in a Japanese boy with recurrent acute liver failure. Researchers led by Junko Matsuda from Kawasaki Medical School, Okayama, Japan, searched for the genetic cause of a young boy’s recurrent episodes of fever-triggered liver dysfunction. They sequenced the entire protein-coding portion of his genome and that of his parents. They found that the boy had inherited two defective copies of the *NBAS* (neuroblastoma amplified sequence) gene, one from each parent. Laboratory experiments indicated that these mutations impaired the ability of the protein encoded by *NBAS* to function correctly. The authors recommend testing for *NBAS* mutations in any children with unexplained liver problems, and then treating with fever-reducing therapies to prevent future life-threatening episodes of liver failure.

## Introduction

Acute liver failure (ALF) is a life-threatening emergency in childhood. Although ~25% of ALF cases are caused by inborn errors of metabolism, the underlying cause remains unknown in ∼50% of cases^1–3^. Recently, biallelic mutations in the neuroblastoma amplified sequence (*NBAS*) gene were identified as a new cause of infantile liver failure syndrome-2 (ILFS2, OMIM 616483)^4^. ILFS2 is an autosomal recessive genetic disorder connected with recurrent episodes of ALF triggered by febrile infection. Previously, the homozygous *NBAS* mutation c.5741G>A (p.Arg1914His) was reported to be associated with a syndrome comprising short stature with optic nerve atrophy and Pelger-Huët anomaly (SOPH) syndrome, which occurs in the genetically isolated Yakut population (OMIM #614800)^5^. Increasing evidence has indicated a phenotypic spectrum of *NBAS* mutation-based disease, from isolated fever-triggered recurrent ALF to a multisystemic disease with short stature, skeletal dysplasia, immunological abnormalities, and optic nerve atrophy resembling SOPH syndrome^6–18^. Because early antipyretic therapy and induction of anabolism have effectively ameliorated the course of ALF with the *NBAS* mutation, it is important to differentially diagnose *NBAS* mutation-based disease. Here, we describe a 3-year-old Japanese boy who presented with recurrent episodes of ALF and carries novel compound heterozygous missense mutations in *NBAS*.

## Materials and methods

### Subjects

After informed consent and the approval of the appropriate institutional ethics review board, peripheral blood samples were obtained from the patient, his parents, and his younger brother, and skin fibroblasts were obtained from the patient. Genomic DNA was extracted from peripheral blood leukocytes.

### Whole-exome sequencing

Exomes of the patient and his parents were captured from the genomic DNA using the Agilent SureSelect Human All Exon V5 kit (Agilent Technologies, Santa Clara, CA, USA) and were sequenced (paired-end, 2 × 124 bp) using the Illumina HiSeq 2500 (Illumina, San Diego, CA, USA). Read alignment to the human reference genome hg19 was performed with Burrows-Wheeler Aligner (http://bio-bwa.sourceforge.net/), and ANNOVER was used for annotation. Germline mutations were detected through our established pipeline, as previously reported (http://genomon.hgc.jp/exome/en/index.html)^19^. Population frequencies in the Human Genetic Variation Database (HGVD), db single-nucleotide polymorphisms (SNP) 131, the Integrative Japanese Genome Variation Database (iJGVD)^20^, and an in-house SNP database were used to filter common SNPs and to confirm the novelty of each mutation. Suspected pathogenic variations in genes known to be associated with ALF or present in accordance with autosomal recessive or X-linked modes of inheritance were identified as candidate genes.

### Sanger direct sequencing

To validate the two *NBAS* variants detected in exons 12 and 24 by whole-exome sequencing, polymerase chain reaction (PCR)-direct sequencing was carried out using the genomic DNA of the patient, his parents, and his younger brother. PCR amplification was carried out using primers specific for *NBAS* exon 12, (forward 5’-TGGCACCTCTAAAGAGTGTCATT-3’, reverse 5’-CTCACCCTTAAGAGAGTATCATTCTAA-3’) and exon 24 (forward 5’-GAATTAGATTCTGTACTGGAGACTTTT-3’, reverse 5’-TCAAAGTGCATAGAAAATGCTTTA-3’). PCR conditions are available on request. The amplified products were sequenced using an ABI 3130xl DNA Analyzer (Thermo Fisher Scientific, Waltham, MA, USA), and NM_015909 was used as the *NBAS* reference sequence.

### In silico analysis

To predict the protein-damaging effects of the suspected pathogenic variations in *NBAS*, three different software programs were used: Polyphen-2 (http://genetics.bwh.harvard.edu/pph2/), Provean (http://provean.jcvi.org/), and MutationTaster (http://www.mutationtaster.org/).

### Western blot analysis

Patient and control fibroblasts were cultivated in minimum essential medium (MEM) supplemented with 10% fetal bovine serum (FBS) and 1% penicillin-streptomycin at 37 and 40 °C, under 5% CO_2_. For Western blots, cells were collected, washed in phosphate-buffered saline (PBS) and resolved in radioimmunoprecipitation assay (RIPA) buffer [50 mM tris(hydroxymethyl) aminomethane (Tris) base pH 7.5, 150 mM NaCl, 1 mM ethylenediaminetetraacetic acid (EDTA), 1% NP-40, 0.5% sodium deoxycholate, and 0.1% sodium dodecyl sulfate (SDS)] containing protease inhibitor cocktail tablets (Complete Mini; Roche Diagnostics, Mannheim, Germany) at 4 °C for 30 min. Lysates were cleared by centrifugation at 10,000 × *g* for 30 min at 4 °C. Protein concentrations were quantified using the bicinchoninic acid (BCA) method (Pierce BCA Protein Assay Kit; Thermo Fisher Scientific). After dilution in 2 × Laemmli sample buffer (Bio-Rad, Hercules, CA, USA) and incubation at 95 °C for 5 min, 15-μg protein samples were subjected to electrophoresis on an 8% SDS-polyacrylamide gel for NBAS and a 12% SDS-polyacrylamide gel for p31 and β-actin and were electroblotted onto polyvinylidene difluoride membranes (Millipore, Burlington, MA, USA). The membranes were blocked with 5% skimmed milk (Wako Pure Chemical Corporation, Osaka, Japan) in TBST buffer (1 × Tris-buffered saline, 0.1% Tween-20) for 1 h and incubated with primary antibodies (all diluted in 0.5% skimmed milk) against NBAS (Thermo Fisher Scientific, PA5-49534; 1:1000), p31 (Sigma-Aldrich, St. Louis, MO, USA, HPA026851; 1:250), and β-actin (Sigma-Aldrich, A5441; 1:10,000) overnight at 4 °C. Next, the membranes were incubated with horseradish peroxidase (HRP)-conjugated anti-rabbit or anti-mouse secondary antibodies (Jackson ImmunoResearch Laboratories, West Grove, PA, USA; 1:5,000) in TBST, and immunoreactivity was visualized with the Luminate Forte Western HRP substrate (Millipore) according to the manufacturer’s protocol, and chemiluminescence signals were digitized using an Amersham Imager 600 (GE Healthcare, Little Chalfont, UK). The β-actin expression levels of each protein sample served as loading controls. Densitometric measurement of the bands on the Western blots was performed using ImageQuant TL 8.1 1 (GE Healthcare). Data were pooled from three independent experiments.

## Results

### Patient clinical presentation

**Fig. 1:**
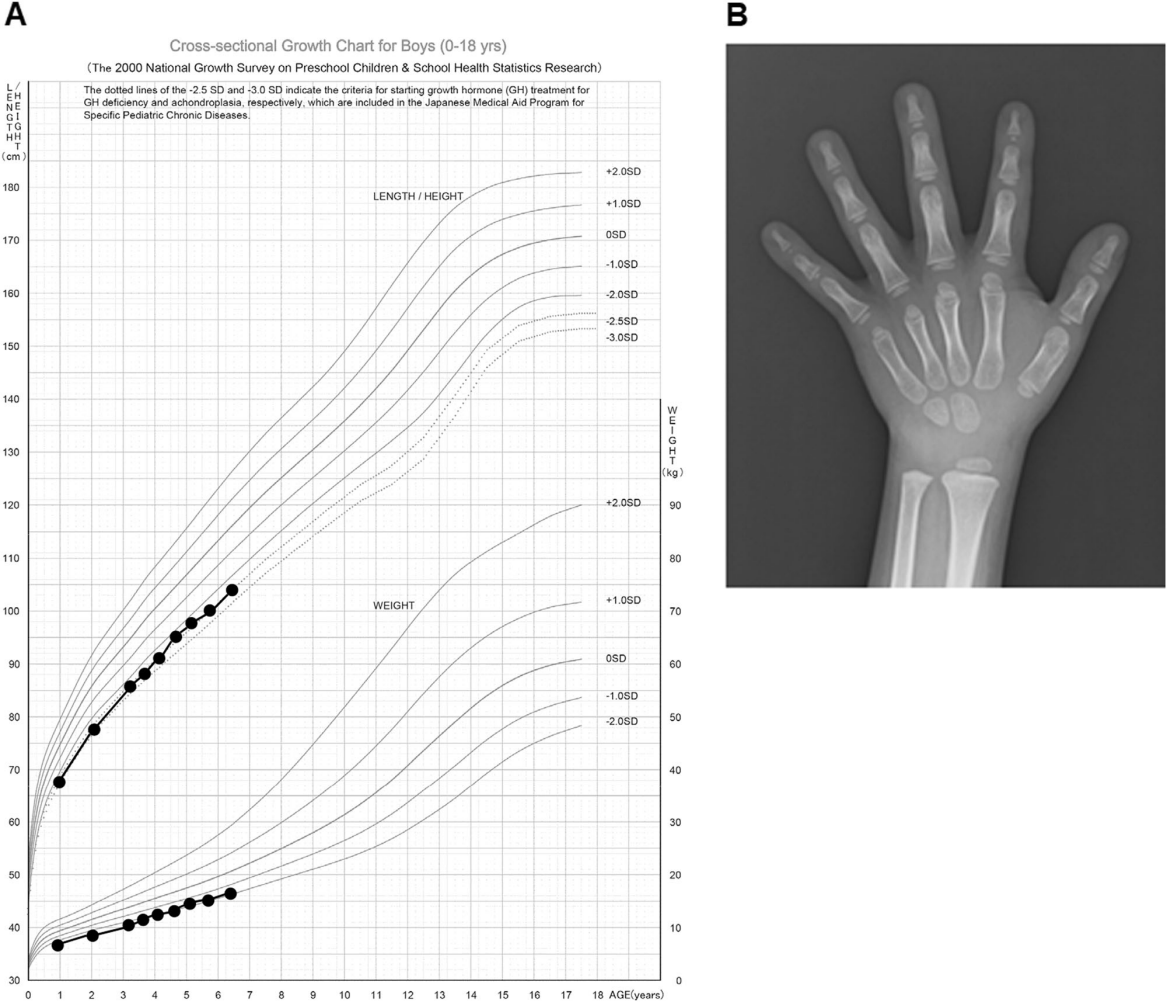
Growth history of the patient. **a** Growth curve showing the persistent short stature of the patient. **b** Hand radiographs at the age of 7 years showing delayed bone maturation by 3 years

The patient was a Japanese boy born to healthy nonconsanguineous parents at 34 weeks gestation due to a threatened premature delivery. The pregnancy was complicated by fetal growth retardation due to the mother’s pregnancy-associated hypertension. The birth weight was 1512 g (−2.3 SD), and the height was 42.5 cm (−0.8 SD). During the neonatal period, gallstones were noticed and treated with ursodeoxycholic acid. The patient’s psychomotor development was normal. He showed short stature: −2.4 SDs in height at the age of 7 years (Fig. 1a). Hand radiographs revealed delayed bone maturation by 3 years (Fig. 1b). There was no family history of liver disease.

**Fig. 2:**
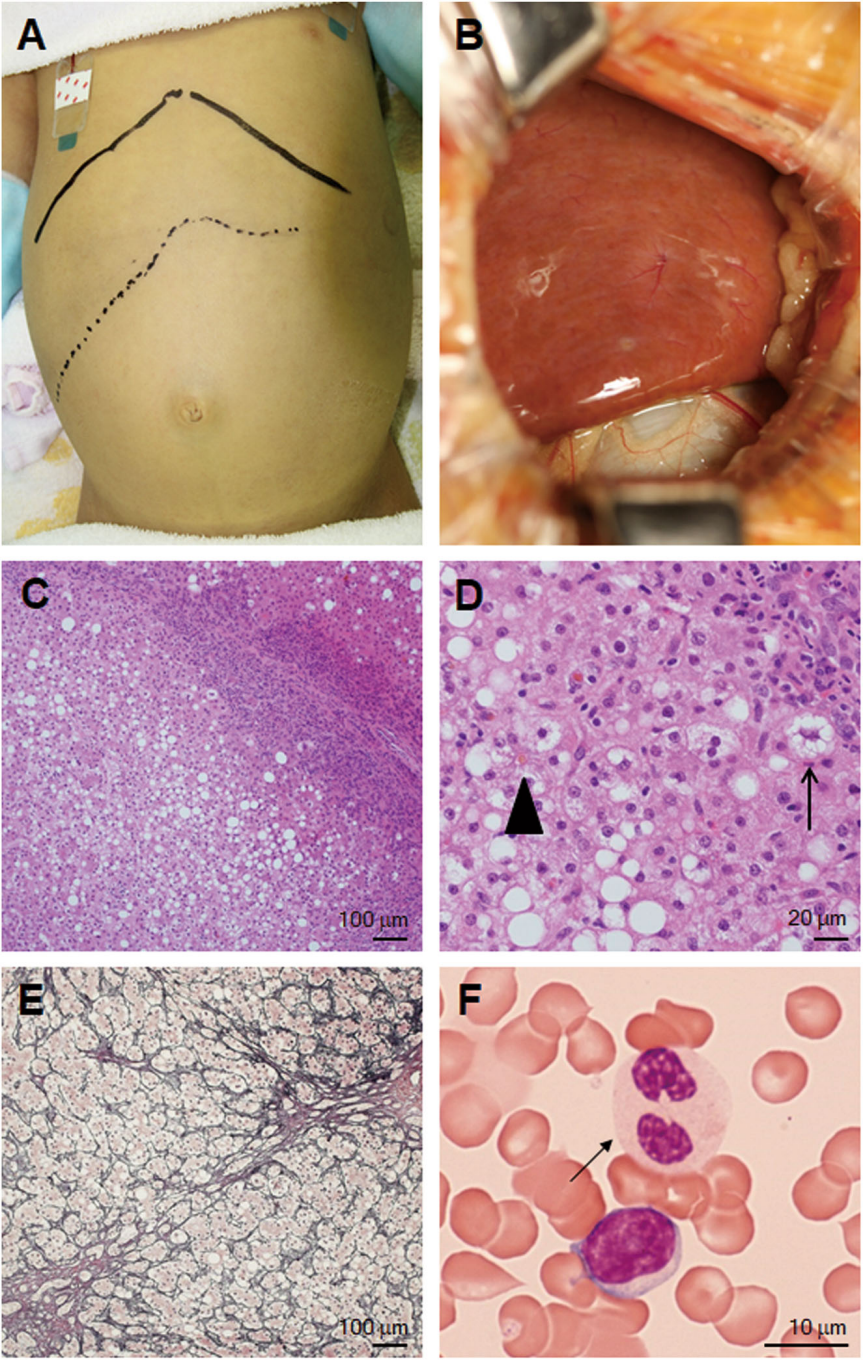
Abdominal appearance and macroscopic and histopathological findings in the liver and peripheral granulocytes. Photograph of the abdomen showing jaundice and hepatomegaly (**a**). Macroscopic findings of the liver showed a yellowish appearance and slightly irregular surface (**b**). Histopathologically, hematoxylin and eosin staining showed moderate parenchymal steatosis and infiltration of inflammatory cells in the portal area (**c**), microvesicular small lipid droplets in the hepatocytes (arrow), and canalicular bile plug formation (arrowhead) (**d**). Reticulin staining showed bridging fibrosis (**e**). Pelger-Huët anomaly in the peripheral granulocytes (arrow) (**f**)

At the age of three years and four months, the patient developed an upper respiratory tract infection with 39 °C fever, cough, and loss of appetite. On day 5 of his fever, he became lethargic and agitated and was then admitted to our hospital. He had jaundice with an enlarged liver palpable 4 cm below the costal margin (Fig. 2a). Laboratory results revealed greatly elevated liver enzyme levels (aspartate aminotransferase [AST], 11,023 U/L [normal range: 7–42]; alanine aminotransferase [ALT], 7422 U/L [normal range: 10–35]; lactate dehydrogenase [LDH], 4968 U/L [normal range: 120–240]); γ-glutamyl transpeptidase [γGT], 37 U/L [normal range: 5–60] and cholinesterase [ChE], 239 U/L [normal range: 222–448]). Total serum bilirubin (4.4 mg/dL [normal range: 0.3–1.2]) and total bile acid (267.5 μmol/L [normal range: < 10]) were significantly increased. Severe coagulopathy (prothrombin time-international normalized ratio (PT-INR), 1.62 [normal range: 0.88–1.12], activated partial thromboplastin time (APTT), > 100 s [normal range: 26.1–35.8]) was observed. Serum creatinine (Cre) and blood urea nitrogen (BUN) were elevated at 2.83 mg/dL (normal range: 0.6–1.1) and 66 mg/dL (normal range: 8–22), respectively, suggesting renal dysfunction. Creatine kinase (CK) was normal (196 mg/dL [normal range: 54–324]). The white blood cell count was elevated and shifted to the left, at 17,820/μL (Stabbed 26%, Segmented 21%), and the C-reactive protein (CRP) (7.15 mg/dL) and serum ferritin (2242 ng/mL [normal range: 30–200]) levels were elevated. His abdominal ultrasound showed severe hepatomegaly without splenomegaly. Electroencephalogram (EEG), magnetic resonance imaging of the brain, electrocardiogram (ECG), and echocardiography were all normal. He was diagnosed as having ALF with renal failure and was managed with intensive treatment, including plasmapheresis, hemodialysis, intravenous antibiotics, intravenous immunoglobulin, and glucose infusion. Following treatment, he made a full recovery and was discharged on the 47th day of disease.

Two months later, at the age of 3 years and 6 months, the patient was readmitted with a second episode of ALF following a sore throat for 2 days and untreated 39 °C fever. Laboratory tests revealed marked elevation of liver enzymes with coagulopathy and renal failure (AST, 22,291 IU/L; ALT, 7666 IU/L; PT-INR, 5.51; APTT, 73.6 s; Cre, 4.64 mg/dL; BUN, 83 mg/dL; CRP, 7.15 mg/dL; ferritin, 7668 ng/mL). Other laboratory data showed changes similar to those of his first ALF episode. Again, complete recovery was achieved following conservative treatment. He was neurologically normal, and there was no impact on his daily life.

Metabolic evaluations during both liver crises showed hyperammonemia (201 μg/dL [normal range: 12–66]), hypoglycemia (6 mg/dL), and metabolic acidosis (blood gas analysis [venous]: pH 7.216, pCO_2_ 42.8 mmHg, bicarbonate 17.0 mmol/L, base excess −10.4 mmol/L). There was a slight elevation of lactate, at 22.8 mg/dL, but a normal level of pyruvate, 0.67 mg/dL. Serum ketone bodies were elevated (total ketone bodies, 589 μmol/L [normal range: 26–122]; acetoacetate, 371.9 μmol/L [normal range: 13–69]; 3-hydroxybutyrate, 217 μmol/L [normal range: <76]). Serum amino acid analysis showed elevations in various amino acids, including valine, leucine, methionine, citrulline, phenylalanine, and arginine, suggestive of the secondary effects of substantial liver dysfunction. Free carnitine level was also elevated. Acylcarnitine profiles showed elevations in C0, C2, C4, C5:1, C5DC, C6, C10DC, and C12DC. Nonketotic dicarboxylic aciduria was noted in the urinalysis for organic acids, including adipate, suberate, 3-hydroxy-adipate, 3-hydroxy-sebacate, dodecanedioic acid, and 3-hydroxy-dodecanedioic acid. Increased excretion of 2-hydroxy sebacate and tyrosine metabolite *p*-hydroxyphenyllactate (PHPLA) was also noted. All of these changes in metabolic biomarkers normalized between the liver crises. Very long-chain fatty acids in the plasma were normal. Ceruloplasmin, copper, and α1-antitrypsin were all normal. The activities of the mitochondrial respiratory chain complex enzymes I, II, III, and IV were all normal in both the liver tissue and skin fibroblasts, indicating that a mitochondrial disorder was not the etiology of the episodes of ALF.

The workups for the infectious agents Epstein-Barr virus, cytomegalovirus, herpes simplex virus, hepatitis A virus, hepatitis B virus, and hepatitis C virus were all negative. The immunological workup, including autoantibodies, serum immunoglobulin levels, complement C3 and C4 levels, CD8^+^ T cell count and function, CD19^+^ B cell count, and natural killer cell count, were all unremarkable. The serum cytokine profile at the time of the liver crisis showed the broad elevation of IL-6, neopterin, sTNF-RI, and sTNF-RII, IL-18, but most prominently IL-6 and neopterin, suggesting a severe cytokine storm during the crisis. Bone marrow aspiration at the time of the second liver crisis revealed hypercellular marrow with occasional hemophagocytosis with Pelger-Huët anomaly in the granulocytes. A repeated peripheral blood smear showed Pelger-Huët anomaly in approximately 5% of granulocytes even in the afebrile and asymptomatic states (Fig. 2f).

A liver wedge biopsy was performed shortly after the second ALF crisis. Grossly, the liver showed a yellowish appearance with a slightly irregular surface (Fig. 2b). Histopathologically, the hepatocytes showed patchy degeneration with cytoplasmic vacuolization and moderate macrovesicular steatosis (Fig. 2c). A few microvesicular lipid droplets and bile plug formation were also seen (Fig. 2d). Infiltration of inflammatory cells, mainly CD8^+^ T cells, was found in the portal area. At the interface of the portal and parenchymal compartments, a ductular reaction was prominent (Fig. 2c). Reticulin staining revealed minimal bridging fibrosis (Fig. 2e). Accumulations of iron and copper were not found by Berlin blue, rhodanine, and rubeanic acid staining (not shown).

### Genetic analyses

**Fig. 3:**
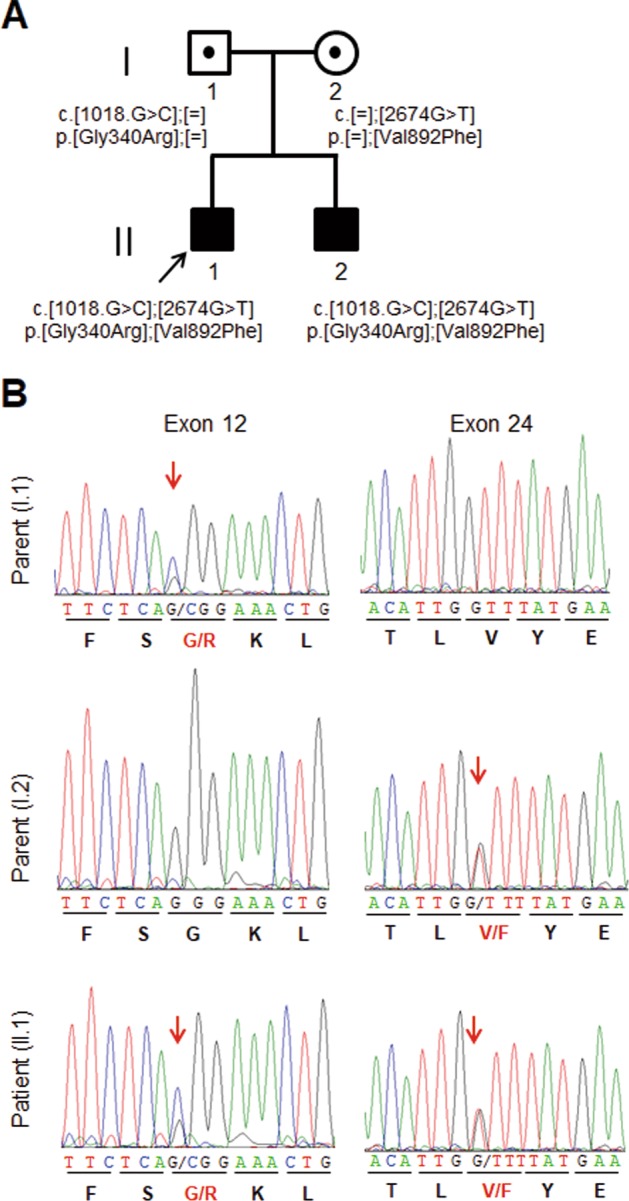
Identification of compound heterozygous mutations in the NBAS gene in the patient and his family. **a** Family pedigree. The patient’s younger brother (II.2) has the same biallelic mutation in the *NBAS* gene. **b** Sanger sequencing chromatograms confirming the segregation of two *NBAS* missense mutations: c.1018G>C (p.Gly340Arg) and c.2674G>T (p.Val892Phe) in this family

Because no etiological diagnosis was reached despite the immunological and metabolic screening, whole-exome sequencing was performed in the patient and in his parents. In total, 25,853 single-nucleotide variants were called. The candidate variations (*n* = 326) were selected by excluding those with (i) ambiguous (unknown) single-nucleotide variations (SNVs) (*n* = 572); (ii) variants only present in unidirectional reads (*n* = 1702); (iii) variant allele frequency <0.25 (*n* = 1593) and (iv) known variants listed in SNP databases (*n* = 21,385). From 326 candidate variations, two heterozygous sequence variants in the *NBAS* gene, chr2:15,629,083C>G (c.1018G>C:p.Gly340Arg) (exon 12) and chr2:15,557,740C>A (c.2674G>T:p.Val892Phe) (exon 24), were detected in the patient. Parental genotyping revealed the c.1018G>C mutation in the heterozygous state in the father and the c.2674G>T mutation in the heterozygous state in the mother, indicating compound heterozygosity of the *NBAS* variants in the patient. Both missense variants were validated by Sanger sequencing (Fig. 3). Neither mutation was present in >120,000 alleles from the Exome Aggregation Consortium (ExAC) Server, and they affect highly conserved amino acid residues. In iJGVD, the largest Japanese genetic variation database^20^, chr2:15,557,740C>A (c.2674G>T:p.Val892Phe) was not identified, and the allele frequency of chr2:15,629,083C>G (c.1018G>C:p.Gly340Arg) was 0.0001 (*n* = 1/7,102).

**Fig. 4:**
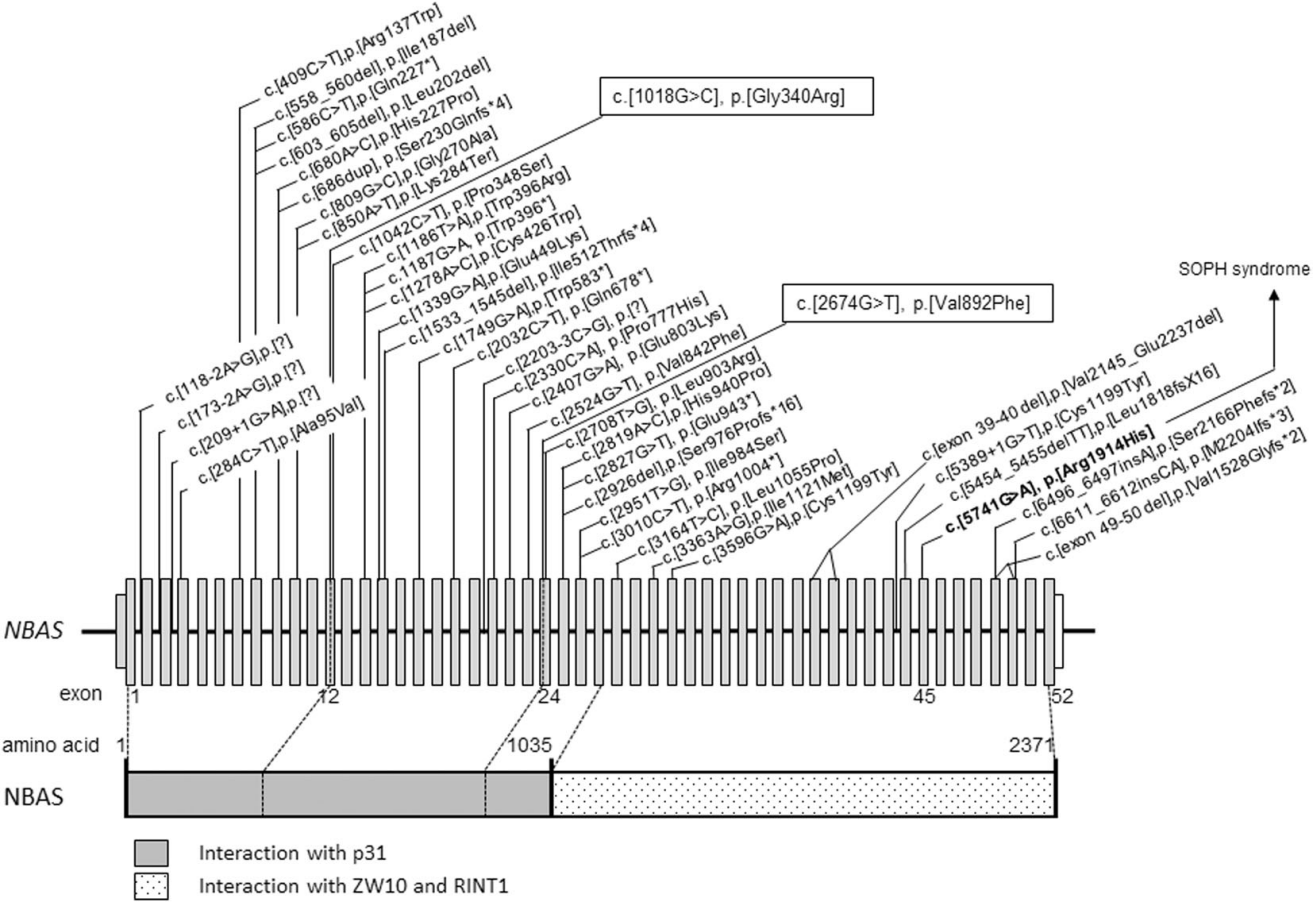
Gene and protein structures of NBAS and identified mutations. Schematic gene and protein structures of NBAS showing the p31 interaction site at the N-terminus and the ZW10 and RINT1 interaction site at the C-terminus. Mutations associated with acute liver failure (ALF), including those in our patient, were mapped to the N-terminal end, whereas mutations associated with SOPH syndrome or related diseases without the liver phenotype were mapped near the C-terminal end. Mutations identified in this study are boxed

In silico analyses predicted these variants to be functionally relevant (PolyPhen 2: probably damaging [1.000]; Provean: deleterious [−7.035 (p.Gly340Arg), −3.838 (p.Val892Phe)]; MutationTaster: disease-causing). Neither *NBAS* mutation had been previously reported in cases of ILFS type 2 or SOPH syndrome. Genetic diagnosis was carried out on the patient’s 2-year-old younger brother to evaluate the possibility of a presymptomatic state of the *NBAS* mutation-based disease. He was identified as carrying the biallelic mutations in *NBAS* (Figs. 3, 4).

### Western blot analysis of NBAS and p31

**Fig. 5:**
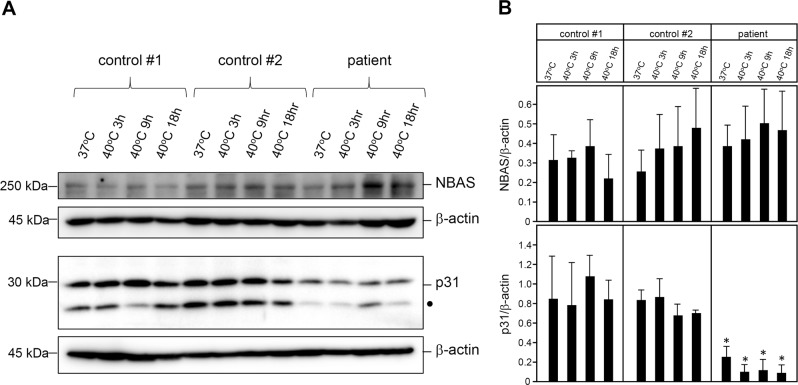
Western blot of NBAS and p31 in cultured fibroblasts from the patient. **a** NBAS protein levels were not decreased even after a shift in the culturing temperature from 37 to 40 °C; however, p31 protein levels were significantly decreased compared to the control subjects. β-actin was used as a loading control. The 15-kDa bands in the p31 western blot indicated by filled circles were isoforms of p31. A typical example of three repeated experiments is shown. **b** Quantification of NBAS and p31 protein levels. In each case, *n* = 3, and the mean ± standard deviation (SD) is shown. * denotes *P* *<* 0.01 compared to the controls

The NBAS protein levels in the fibroblast sample from the patient were not decreased, even after changing the culturing temperature from 37 to 40 °C; however, p31 protein levels were significantly reduced (Fig. 5). As p31 forms the SNARE complex with NBAS, mutations in *NBAS* may impair NBAS–p31 interactions, resulting in a loss of protein stability of p31 followed by dysregulation of Golgi-to-endoplasmic reticulum (ER) retrograde vesicular trafficking in the patient.

## Discussion

*NBAS* mutations were first identified in SOPH syndrome patients, but they do not commonly exhibit a liver phenotype^5^. In 2015, Haack et al.^4^ identified homozygous or compound heterozygous *NBAS* gene mutations in German patients with recurrent ALF. More than 15 existing studies on cases carrying homozygous or compound heterozygous *NBAS* gene mutations revealed the broad phenotypic spectrum of *NBAS* mutation-based diseases, ranging from isolated recurrent ALF to a multisystemic disease with short stature, skeletal dysplasia, facial dysmorphism, immunological abnormalities, Pelger-Huët anomaly, and optic nerve atrophy resembling SOPH syndrome (Fig. 4)^4–18^. The extrahepatic phenotypes observed in our patient were Pelger-Huët anomaly and short stature. He did not present with optic atrophy, facial dysmorphism, or skeletal dysplasia.

The liver phenotype in *NBAS* mutation-based disease is most typically recurrent ALF triggered by febrile infections, but mild elevations of liver biochemical values occur in some cases^4, 6–14, 16, 17^. These elevations often develop during infancy or early childhood and become less frequent with age. Coagulopathy and jaundice were observed in most of the cases. Secondary metabolic abnormalities, including hypoglycemia and hyperammonemia, were often observed. Complete recovery is typically achieved with antipyretic therapy and induction of anabolism, including parenteral glucose and lipids. Our patient developed ALF twice at the age of 3 years, both episodes triggered by febrile infections. He showed severe coagulopathy with renal failure but recovered completely with conservative treatment. After the diagnosis of *NBAS* mutation-based disease, he suffered multiple hospitalizations with febrile illnesses associated with liver dysfunction; however, early antipyretic therapy using intravenous injection of acetaminophen and glucose infusion prevented further occurrences of ALF until the age of seven. His younger brother was found to carry the same mutations as the proband’s at the age of 2 years. He shared only Pelger-Huët anomaly in peripheral granulocytes as a symptom of *NBAS* mutation-based disease. After the genetic diagnosis, he has received early antipyretic therapy and has never experienced fever-triggered ALF or liver dysfunction to date (he is currently 5 years old). These findings indicate the possible effectiveness of early antipyretic therapy to prevent life-threatening ALF. However, it is also possible that there is a phenotypic heterogeneity of the affected organs among the family members. Further investigation to reveal the penetration and phenotype-genotype correlation in *NBAS* mutation-based disease is awaited.

The molecular pathogenesis by which NBAS contributes to liver disease and to fever dependency is not fully understood. The NBAS protein is considered to be involved in Golgi-to-ER retrograde vesicular trafficking and to control nonsense-mediated mRNA decay^21–23^. The NBAS protein interacts directly with p31, ZW10, and RINT1 and is believed to play a primary role in SNARE assembly at the ER^21^. It is proposed that the site at amino acid positions 1–1035 is the p31 binding site. Mutations associated with ALF, including those in our patient, are mostly found at the N-terminal end of *NBAS* (Fig. 4). In contrast, the p.Arg1914His mutation carried by SOPH-affected Yakut individuals in the homozygous state is located near the C-terminal end and does not cause ALF^5^. Our patient’s skin fibroblasts showed a decreased p31 protein level (Fig. 5). Thermal susceptibilities of NBAS and p31 proteins have been reported previously^4, 8^. These findings suggest that the loss of the NBAS-p31 interaction may cause alterations to Golgi-to-ER retrograde vesicular trafficking, which leads to liver dysfunction, especially in the febrile condition^8^. Further in-depth mechanistic studies are needed to establish the genotype-phenotype correlations and to reveal the wide phenotypic spectrum of *NBAS* mutation-based disease. Further investigations using the patient’s iPS cell-derived hepatocytes will show whether the episodes of liver disease are due to alterations in Golgi-to-ER retrograde vesicular trafficking and/or impaired nonsense-mediated mRNA decay, or a yet-to-be-discovered NBAS function.

We describe the first reported Japanese case of fever-triggered recurrent ALF carrying novel compound heterozygous missense mutations of *NBAS*. We recommend sequencing *NBAS* in any patients with unexplained recurrent ALF, especially in individuals with fever-associated hepatic dysfunction.
